# Serum Neutrophil Gelatinase-Associated Lipocalin (NGAL) in HCV-Positive Egyptian Patients Treated with Sofosbuvir

**DOI:** 10.1155/2020/1632959

**Published:** 2020-01-27

**Authors:** Ali Nada, Mohamed Abbasy, Aliaa Sabry, Azza Mohamed Abdu Allah, Somaia Shehab-Eldeen, Nada Elnaidany, Hanan Elimam, Kawthar Ibraheem Mohamed Ibraheem, Abdallah Essa

**Affiliations:** ^1^Hepatology Department, National Liver Institute, Menoufia University, Shebin-Elkom, Egypt; ^2^Biochemistry and Molecular Biology Department, Faculty of Medicine, Menoufia University, Shebin-Elkom, Egypt; ^3^Tropical Medicine Department, Faculty of Medicine, Menoufia University, Shebin-Elkom, Egypt; ^4^Faculty of Pharmacy, MSA University, 6th of October City, Egypt; ^5^Biochemistry Department, Faculty of Pharmacy, University of Sadat City, Sadat City, Egypt; ^6^Medical Microbiology and Immunology Department, Faculty of Medicine, Ain Shams University, Cairo, Egypt

## Abstract

**Background:**

Direct-acting antivirals (DAAs) made a drastic change in the management of HCV infection. Sofosbuvir is one of the highly potent DAAs, eliminated mainly through the kidney. But concerns about renal safety during treatment may limit its use. Neutrophil gelatinase-associated lipocalin (NGAL) has been proven as a predictor of renal tubular injury. Hence, the aim of this work was to assess serum neutrophil gelatinase-associated lipocalin (NGAL) in HCV-positive patients before and after treatment with the sofosbuvir-based antiviral regimen.

**Methods:**

This prospective study included 87 Egyptian patients with chronic HCV infection treated with sofosbuvir plus daclatasvir with or without ribavirin for 12 weeks. Serum NGAL was measured before and at the end of treatment (EOT). Analysis of NGAL and estimated glomerular filtration rate (eGFR) evolution was done.

**Results:**

Our results showed a statistically significant decrease in serum NGAL (*P*=0.02) with a nonsignificant reduction in eGFR (*P*=0.02) with a nonsignificant reduction in eGFR (*P*=0.02) with a nonsignificant reduction in eGFR (*P*=0.02) with a nonsignificant reduction in eGFR (*P*=0.02) with a nonsignificant reduction in eGFR (

**Conclusions:**

Sofosbuvir appears to have no nephrotoxic effects and is safe to treat patients with chronic HCV infection.

## 1. Introduction

Chronic hepatitis C virus (HCV) infection affects over 170 million people globally, and it is considered a challenging public health problem in Egypt [1, 2]. Incidence of renal insufficiency is higher by around 40%, in HCV infected individuals compared to HCV negative individuals. This renal insufficiency ranges from mild to end-stage renal disease and usually complicates the prognosis and treatment of HCV infection [3].

The development of newer and more effective direct-acting antivirals (DAAs) can significantly decrease the burden of hepatitis C, with possible elimination of this virus [4]. But, concerns on renal safety may limit its use in HCV infected patients despite its documented efficacy [5].

Sofosbuvir (SOF) was the first-in-class NS5B HCV Nucleotide Polymerase Inhibitor approved in December 2013 [6]. It is one of the highly effective and widely used DAAs. It is used in combination with other DAAs, such as daclatasvir, simeprevir, or ribavirin [7].

Sofosbuvir elimination is mainly by renal clearance, so its concentration increases in patients with severe renal insufficiency. International guidelines recommend that adjustments of DAAs including sofosbuvir are not required for patients with an estimated glomerular filtration rate (eGFR) of ≥30 ml/min [8].

In clinical practice, creatinine and estimated glomerular filtration rate (eGFR) are the only parameters used for monitoring the renal safety during HCV treatment. In fact, eGFR may be relatively inaccurate as a single measure of renal insufficiency in conditions like acute kidney disease, high GFR, and liver cirrhosis [9]. So, Kidney Disease Improving Global Outcome (KDIGO) guidelines advocate using complementary biomarkers of renal damage, beside serum creatinine [10].

Neutrophil gelatinase-associated lipocalin (NGAL) is a promising biomarker for injury of the renal tubules. It is a protein produced in the renal epithelium in response to nephron injury. It is used to diagnose acute kidney injury in different settings (e.g., sepsis, after contrast agent exposure, and after surgery) [11].

This study aimed to assess the serum level of NGAL in patients treated with sofosbuvir-based regimen, which could reflect the effects of sofosbuvir on the renal tubules.

## 2. Methods

### 2.1. Patients

Our prospective study included 87 Egyptian patients with chronic hepatitis C infection who attended the outpatient clinic of the National Liver Institute in Shebin-Elkom (Egypt) from January 2018 to January 2019. Inclusion criteria were HCV-positive patients prescribed daily treatment with sofosbuvir-based DAAs (sofosbuvir 400 mg plus daclatasvir 60 mg) with or without ribavirin (RBV) for 12 weeks. Exclusion criteria were human immunodeficiency virus (HIV) coinfection, Hepatitis B surface antigen positivity, leukocytosis (total leukocyte count > 12000 cells/*μ*l), primary kidney diseases, patients with eGFR < 30 ml/min, and ongoing or history of previous HCV therapy.

All patients provided informed written consent before enrollment, and the study was approved from the Ethical Committee of the National Liver Institute and was done with respect to the Declaration of Helsinki.

All participants were subjected to thorough medical history taking complete physical and ultrasonic abdominal examination. Routine laboratory tests including ALT, AST, ALP, total bilirubin, serum albumin, INR, complete blood count (including neutrophil and platelet count), fasting blood glucose (FBG), blood urea, and serum creatinine were performed, in addition to hepatitis B serology and HCV quantitative PCR.

All these exams and investigations were repeated again after the end of treatment (EOT).

### 2.2. Calculations

Estimated glomerular filtration rate (eGFR) was calculated using Chronic Kidney Disease Epidemiology Collaboration (CKD-Epi) equation (141 × min (SCr/*K*, 1)^*α*^ × max (SCr/*K*, 1)^−1.209^ × 0.993^Age^ × 1.018 (if female) × 1.159 (if black)). SCr is serum creatinine (mg/dL), *K* is 0.7 for females and 0.9 for males, *α* is −0.329 for females and −0.411 for males, min indicates the minimum of SCr/*K* or 1, and max indicates the maximum of SCr/*K* or 1. We used this equation as it is more accurate for eGFR ≥60 ml/min [10].

Fibrosis four (FIB4) score or AST to platelet ratio index (APRI) were used to estimate liver fibrosis before treatment. The following formula was used for calculation of FIB4: age (years) × AST (IU/L)/(platelets (10^9^/L) × ALT^1/2^ (IU/L)), and the following formula was used for APRI score calculation: (AST (IU/L)/AST upper normal limit (IU/L))/platelets (10^9^/L) × 100, in which the AST upper normal limit was fixed at 40 IU/L [12, 13].

Patients were ranked at baseline according to KDIGO-CKD classification into stage 1 (eGFR ≥ 90 ml/min), stage 2 (eGFR 60–89 ml/min), and stage 3a (eGFR < 60 ml/min).

### 2.3. Sampling

Under complete aseptic conditions, 8 ml venous blood sample was collected from each patient after 8 hours of fasting. In a sterile tube, 4 ml was taken, allowed to clot, and then centrifuged for 15 minutes at 3000 rpm for separation of the serum to assess all serum biochemical tests which include AST, ALT, total bilirubin, albumin, creatinine, fasting blood glucose (FBG), and neutrophil gelatinase-associated lipocalin (NGAL). For the remaining 4 ml of blood, 2 ml was taken in the EDTA tube, for assessing hemoglobin, leukocytes, and platelets count, in addition to HCV quantitative PCR, and 2 ml was taken in citrated tubes for assessing INR. After 12 weeks of treatment, all sampling procedures were repeated.

### 2.4. Laboratory Methods

On Synchron CX9 autoanalyzer, biochemical tests for measurement of AST, ALT, total bilirubin, albumin, creatinine, and FBG were done utilizing kit supplied by Beckman (Beckman Instrument. Inc. Fullerton, California, USA). Hepatitis viral marker (HCV-Ab) was done by “ECLIA” utilizing Cobas 411 analyzers (Roche Diagnostics, Germany), and QIAGEN viral RNA Mini Extraction Kit was used in nucleic acid extraction for RT-PCR for HCV.

### 2.5. NGAL Measurement

Assay of serum NGAL was done by the Enzyme-Linked Immune Sorbent Assay (ELISA) method, using the kit supplied by Shanghai Sunred Biological Technology Co., Ltd. Catalogue No. 201-12-1720. The kit uses a double-antibody sandwich enzyme-linked immunosorbent assay (ELISA). Neutrophil gelatinase-associated lipocalin (NGAL) was added to monoclonal antibody enzyme well which is precoated with human neutrophil gelatinase-associated lipocalin (NGAL) monoclonal antibody incubation; then, neutrophil gelatinase-associated lipocalin (NGAL) antibodies labeled with biotin and combined with streptavidin-HRP were added to each other to form immune complex; then for removal of the uncombined enzyme, incubation and washing again were done. Then, chromogen solutions A and B were added. The liquid color changes into blue, and finally, the color becomes yellow at the effect of acid. The chroma of color was positively correlated to the concentration of the Human Substance Neutrophil Gelatinase-Associated Lipocalin (NGAL) in the sample.

### 2.6. Statistical Analysis

Quantitative data were presented as mean and standard deviation, while qualitative data were presented as number and percentage. Comparison of normally distributed quantitative data was done using Student's *t*-test, while the Wilcoxon–Mann–Whitney test was used for nonparametric data. Comparison of qualitative variables was done using the *χ*2 test. Correlation between NGAL and other variables was done using Spearman correlation coefficients. These statistical analyses were performed using SPSS Statistics 21. A *P* value <0.05 was considered statistically significant.

## 3. Results

Eighty-seven HCV RNA positive patients were included in our study. They were 26 males (29.9%) and 61 females (70.1%) with a mean age of 50.18 ± 10.01. The main characteristics of the studied population are shown in Table 1.

**Figure 1:**
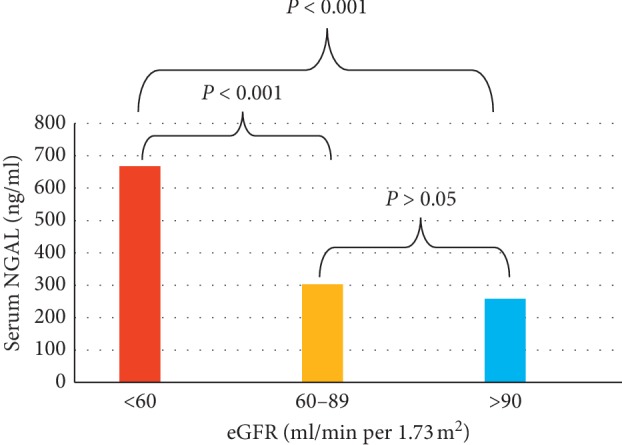
Serum neutrophil gelatinase-associated lipocalin (NGAL) at baseline according to ranks of the estimated glomerular filtration rate.

By ranking the patients according to different parameters like gender, diabetes mellitus, hypertension, cirrhosis, viral load, and FIB4 and APRI scores, we did not find any significant difference, as shown in Table 2, but by ranking patients by eGFR at baseline, serum NGAL was significantly higher in patients with eGFR less than 60 ml/min per 1.73 m^2^ (667.44 ± 687.43 ng/ml) compared to patients with eGFR 60–89 ml/min per 1.73 m^2^ (302.85 ± 169.45 ng/ml), or patients with eGFR more than 90 ml/min per 1.73 m^2^ (258.52 ± 121.02 ng/ml), as shown in Figure 1.

**Figure 2:**
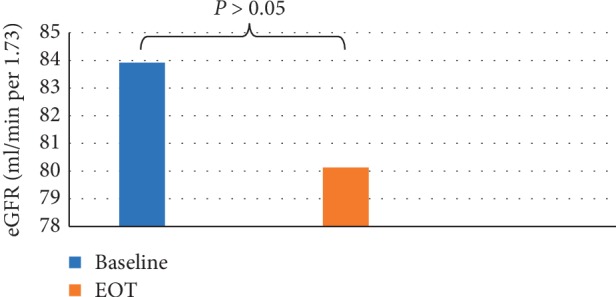
Estimated glomerular filtration rate (eGFR) at baseline and EOT among the overall studied population.

**Figure 3:**
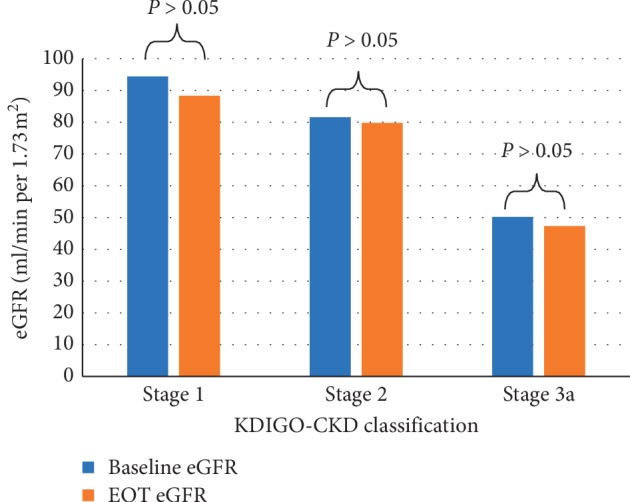
Estimated glomerular filtration rate (eGFR) at baseline and EOT among patients ranked according to KDIGO-CKD classification.

Evaluation of the changes in eGFR in the studied population revealed a statistically nonsignificant change (83.92 ± 20.18 ml/min per 1.73 m^2^ at baseline and 80.13 ± 21.1 ml/min per 1.73 m^2^ at EOT, *P*=0.06), as shown in Figure 2. Furthermore, ranking patients according to KDIGO-CKD classification, eGFR was nonsignificantly reduced by treatment in all KDIGO-CKD stages (*P* > 0.05), as shown in Figure 3.

**Figure 4:**
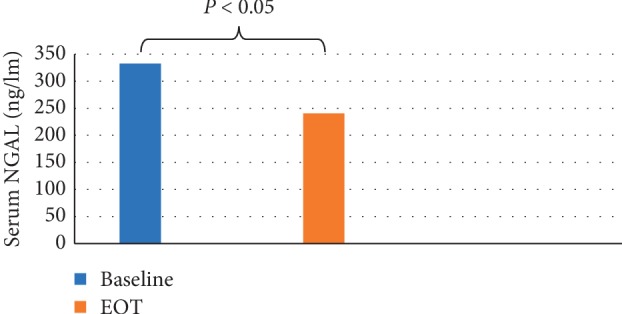
Serum neutrophil gelatinase-associated lipocalin (NGAL) at baseline and EOT among the overall studied population.

**Figure 5:**
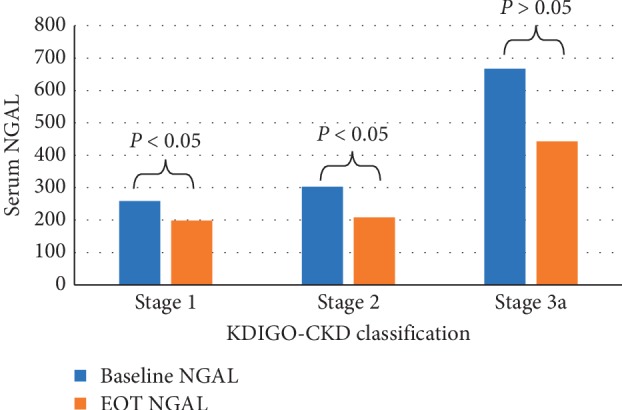
Serum neutrophil gelatinase-associated lipocalin (NGAL) at baseline and EOT among patients ranked according to KDIGO-CKD classification.

Evaluation of the changes in serum NGAL in the overall studied population revealed a statistically significant decrease (332.65 ± 302.36 ng/ml at baseline to 240.57 ± 156.18 ng/ml at EOT, *P*=0.02), as shown in Figure 4. Also, differences in NGAL values (before and after treatment) were evaluated in patients ranked according to KDIGO-CKD classification which revealed a statistically significant decrease in serum NGAL levels in patients with KDIGO-CKD stage 1 (*P*=0.014) and stage 2 (*P*=0.034), while the decrease was statistically nonsignificant in patients with stage 3 (*P*=0.25), as shown in Figure 5.

All patients started sofosbuvir plus daclatasvir, while ribavirin was added to 27 patients. At weeks 4 and 12, HCV RNA was undetectable in all patients.

**Figure 6:**
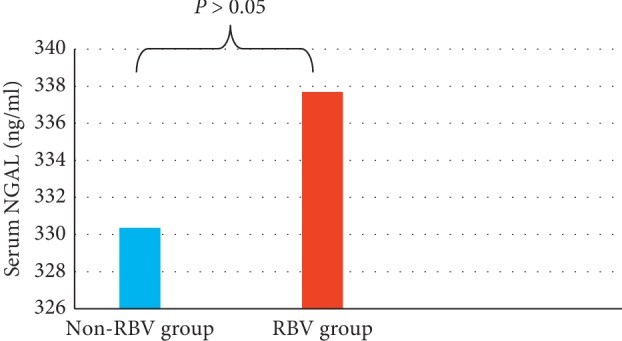
Serum neutrophil gelatinase-associated lipocalin (NGAL) at EOT according to ribavirin (RBV) use.

By ranking patients according to the use of ribavirin (RBV), we did not find any statistically significant difference in serum NGAL at EOT between groups (330.37 ± 221.03 for non-RBV group and 337.69 ± 437.6 for RBV group, *P*=0.71), as shown in Figure 6.

Correlation analysis of serum NGAL at baseline with different baseline parameters did not reveal any significant correlation. Also, correlation analysis of serum NGAL at EOT with all baseline and EOT parameters did not reveal any significant correlation, as shown in Table 3.

## 4. Discussion

Chronic hepatitis C infection is a significant cause of liver cirrhosis and hepatocellular carcinoma. It is also a recognized cause of chronic kidney impairment [14]. The recent availability of direct-acting antivirals (DAAs) has led to a revolution in the treatment of HCV infections, being a highly effective treatment option irrespective of the stage of liver fibrosis [15]. However, there have been concerns about renal function deterioration associated with DAAs [5].

Sofosbuvir (SOF) is a potent nucleoside NS5B polymerase inhibitor. It is mainly eliminated through the kidneys, so its level may increase in patients with severe kidney disease. Recently, concerns about its potential nephrotoxicity have been raised [8].

To date, only one study conducted by Strazzulla et al. attempted to retrospectively investigate the role of NGAL in evaluating the effect of sofosbuvir-based antivirals on renal tubules, in 18 patients with chronic HCV infection. No large prospective studies have been published about this issue [5]. Hence, this study aimed to assess serum NGAL both before and after the twelve weeks of therapy with sofosbuvir-based regimen, in a larger population of HCV RNA-positive patients.

Patients included in our study were treated with sofosbuvir plus daclatasvir, with or without ribavirin. Daclatasvir is metabolized mainly by the liver, and it has been recommended for the treatment of patients with severe renal impairment or end-stage kidney disease [16]. And to the best of our knowledge, no significant data are available on ribavirin nephrotoxicity [17]. Regarding sofosbuvir, international guidelines recommended that its use should be restricted to patients with an estimated glomerular filtration rate (eGFR) ≥ 30 ml/min per 1.73 m^2^. These limitations however need to be considered in the background of the absence of clinical studies evaluating its safety in patients with an eGFR < 30 ml/min per 1.73 m^2^ [18].

By evaluating the changes in eGFR after treatment, we observed a nonsignificant reduction in the overall studied population and in different subgroups ranked by KDIGO-CKD classification.

Strazzulla et al. reported a statistically significant eGFR reduction in the overall studied population and in patients with KDIGO-CKD stage 1 (normal eGFR at baseline). However, multivariate analysis showed that high eGFR at baseline is a predictor of eGFR reduction after treatment, suggesting that this reduction was due to regression to the mean effect rather than due to glomerular injury (5).

Interestingly, our results revealed significant reduction in serum NGAL after twelve weeks of sofosbuvir-based HCV therapy, and a nonsignificant decrease in eGFR. Moreover, analysis of the serum NGAL level in patients ranked by KDIGO-CKD classification showed a significant reduction in relation to different stages; however, this reduction was not statistically significant in patients with moderate renal impairment (stage 3a). These findings suggest that elevated serum NGAL levels before treatment were due to the effect of hepatitis C virus on the renal tubules, and with successful treatment by sofosbuvir-based antiviral therapy, these levels decreased.

Virus-related kidney disease may be attributed to cryoglobulinemia, subendothelial or intraluminal deposits of immune complexes, and possibly a direct cytopathic effect, as HCV core proteins were isolated in both glomerular and tubular tissues [19, 20].

In contrast, Strazzulla et al. observed a significant increase in serum NGAL in patients with chronic HCV infection treated with sofosbuvir plus ledipasvir [5]. They found the same observation in their previous study which was conducted on patients with chronic hepatitis C treated with DAAs other than sofosbuvir-based regimen. They explained that by either drug nephrotoxicity or inflammation due to the activity of antiviral drugs [21].

There was a nonsignificant decrease in the eGFR after treatment, but serum NGAL levels also decreased. We have no explanation for this discordance, but similarly, Strazzulla et al. found that eGFR was below normality around 50%, while NGAL was in the range of normality in most individuals before treatment with DAAs. Moreover, among the six patients with increased NGAL, two cases had normal eGFR, which suggests a discordance between the two methods when interpretation is “categorical.” Despite these findings, they found that plasmatic NGAL was statistically correlated with eGFR in the overall population. They mentioned that it is difficult to explain apparent discrepancies and concluded that further studies should evaluate the rate of concordance between the two methods in diverse stages of liver disease [21].

In the current study, we did not find a significant correlation between serum NGAL and the viral load, suggesting that renal affection in chronically HCV infected patients has no relation to the viral load. Similarly, Strazzulla et al. did not find any correlation between serum NGAL and the burden of HCV RNA [21]. This is supported by previous studies which documented that, even with undetectable HCV RNA, occult hepatitis C may be the cause of a proportion of glomerulonephropathies [22, 23].

Also, we did not find any significant difference in serum NGAL, between cirrhotic and noncirrhotic patients. This agrees with Gungor et al. who did not find differences in serum NGAL between cirrhotic patients and healthy controls although they found that a high plasma NGAL is a predictor of mortality in cirrhotic patients [24].

Our results showed that serum NGAL was significantly higher in patients with eGFR < 60 ml/min per 1.73 m^2^ compared to patients with eGFR 60–90 or >90 ml/min per 1.73 m^2^. Strazzulla et al. reported similar results [21]. In contrast, Alhaddad et al. observed a significantly lower plasma NGAL in HCV-positive cirrhotic patients with eGFR < 60 ml/min compared to cirrhotic patients with eGFR ≥ 60 ml/min [25].

The discrepancies between our study and the study by Strazulla et al. in the context of difference in posttreatment serum NGAL levels could be partly explained by the genetic difference in the study population as well as the genotype of the virus itself (the most common genotype in Egypt is genotype IV as compared to Italy, where the commonest genotype reported is genotype I).

By ranking patients according to the use of ribavirin, we did not find any significant difference between the two groups. Strazzulla et al. found the same observation, which supports that ribavirin has no nephrotoxic effect [5].

The correlation between nephrotoxicity and SOF-based therapy was proposed in a few case reports [26, 27]. However, multiple, large retrospective studies concluded that SOF-based regimen does not cause higher acute kidney injury in HCV patients, when compared to SOF-free treatment, and most of these studies had generally stable eGFR and serum creatinine during treatment [28–30].

Finally, the results of the present study demonstrated that serum NGAL has been decreased after treatment of HCV-positive patients with sofosbuvir-based regimen, without a significant change in eGFR, which could reflect that sofosbuvir has no injurious effect on the renal tubules. Moreover, it possibly has a protective effect on renal tubules by eliminating the HCV-induced renal tubular injury; however, large-scale population studies are highly warranted.

Our study has some limitations. We did not include patients with severe renal impairment. We also did not utilize other reliable markers of renal impairment like proteinuria and cystatin C, and we did not measure ratio of urinary NGAL/serum NGAL, which could be a better marker of renal injury.

## 5. Conclusions

HCV treatment with sofosbuvir-based regimen showed significant decrease in the serum NGAL level after HCV eradication, compared to pretreatment levels, which could reflect a relatively stable renal function while using sofosbuvir.

## Figures and Tables

**Table 1:** Characteristics of the studied population before treatment.

Characteristics	Studied group (*n* = 87)
Qualitative variables (no., %)	
Gender	
Male	26 (29.9%)
Female	61 (70.1%)

BMI	
18.5–24.9	20 (23%)
25.0–29.9	48 (55.2%)
30.0 and above	19 (21.8%)

Diabetes	
Yes	6 (6.9%)
No	81 (93.1%)

Hypertension	
Yes	18 (20.7%)
No	69 (79.3%)

Liver	
Normal	60 (69%)
Cirrhotic	27 (31%)

Quantitative variables (mean ± SD)	
Age (years)	50.18 ± 10.01
HCV RNA (copies/ml)	925507 ± 1241001
Total bilirubin (mg/dl)	0.88 ± 0.2
Serum albumin (mg/dl)	4.32 ± 0.52
INR	1.06 ± 0.08
AST (U/L)	44.66 ± 18.2
ALT (U/L)	49.2 ± 28.97
HB (g/dl)	13.48 ± 1.4
Platelet (*n* × 10^3^/*μ*l)	208.86 ± 66.02
Creatinine (mg/dl)	0.9 ± 0.48
eGFR (ml/min per 1.73 m^2^)	83.92 ± 20.18
FIB4 score	1.7 ± 0.9
APRI score	0.6 ± 0.39

BMI: body mass index; HCV RNA: hepatitis C virus-ribonucleic acid; INR: international normalization ratio; AST: aspartate aminotransferase; ALT: alanine aminotransferase; HB: hemoglobin; eGFR: estimated glomerular filtration rate; FIB4 : fibrosis four; APRI : AST to platelet ratio index.

**Table 2:** Serum NGAL among the patients when ranked by different parameters.

Parameter	(No., %)	Serum NGAL (ng/ml)	*P* value
Gender			
Male	26 (29.9%)	336.69 ± 217.31	0.75
Female	61 (70.1%)	330.92 ± 333.69	

Diabetes			
Yes	6 (6.9%)	265.73 ± 60.40	0.39
No	81 (93.1%)	337.60 ± 312.55	

Hypertension			
Yes	18 (20.7%)	362.4 ± 279.77	0.35
No	69 (79.3%)	324.88 ± 309.44	

Liver			
Normal	60 (69%)	327.19 ± 221.00	0.65
Cirrhotic	27 (31%)	344.78 ± 437.42	

HCV RNA (copies/ml)			
Less than 1000000	51 (58.6%)	336.57 ± 356.83	0.58
More than 1000000	36 (41.4%)	327.09 ± 206.59	

FIB4			
G1: <1.45	41 (47.1%)	329.99 ± 368.24	*P*1=0.95
G2: 1.45 to 3.25	42 (38. 3%)	346.56 ± 240.549	*P*2=0.38
G3: >3.25	4 (4.6%)	213.8 ± 15.05	*P*3=0.12

APRI			
G1: <0.5	45 (51.7%)	329.99 ± 368.24	*P*1=0.23
G2: 0.5–1.5	38 (43.7%)	346.56 ± 240.55	*P*2=0.53
G3: >1.5	4 (4.6%)	213.8 ± 15.05	*P*3=0.67

G: group; *P*1: *P* value for G1 vs G2; *P*2: *P* value for G1 vs G3; *P*3: *P* value for G2 vs G3.

**Table 3:** Correlation analysis of serum NGAL at EOT with baseline and EOT parameters.

Parameter	*r*	*P* value
*Baseline parameters*		
Age (years)	−0.05	0.74
BMI	0.006	0.97
HCV_PCR (copies/ml)	0.26	0.09
Total bilirubin (mg/dl)	0.07	0.66
Serum albumin (mg/dl)	0.11	0.48
INR	−0.02	0.89
ALT (U/L)	−0.09	0.55
AST (U/L)	−0.11	0.47
Creatinine (mg/dl)	−0.04	0.78
eGFR (ml/min per 1.73 m^2^)	0.13	0.39
HB (g/dl)	0.11	0.46
Platelets (*n* × 10^3^/*μ*l)	0.13	0.39
FIB4 score	−0.19	0.21
APRI score	−0.17	0.26

*EOT parameters*		
ALT (U/L)	−0.05	0.72
AST (U/L)	-0.1	0.49
Total bilirubin (mg/dl)	-0.03	0.83
Creatinine (mg/dl)	0.07	0.65
eGFR (ml/min per 1.73 m^2^)	0.09	0.55
HB (g/dl)	0.16	0.06
Platelets (*n* × 10^3^/*μ*l)	0.10	0.50

*r*: correlation coefficient.

## Data Availability

The data used to support the findings of this study are restricted by the Ethical Committee of the National Liver Institute in order to protect patient privacy. Data are available from the first author for researchers who meet the criteria for access to confidential data.
